# Supplementary material for: Impact of Distribution of a Tip Sheet to Increase Early Detection and Prevention Behavior among First-Degree Relatives of Melanoma Patients: A Randomized Cluster Trial
Source: Cancers (Basel). 2022 Aug 10;14(16):3864. doi: 10.3390/cancers14163864 (PMC9406196; doi:10.3390/cancers14163864)
Supplement: Supplementary file 1 [file cancers-14-03864-s001.zip › cancers-1802800-supplementary.pdf]

cancers

MDPI

Figure S1. The French and English version of the tip sheet given to patients intended for their first-degree relatives.

Fiche de conseils de protection solaire et de dépistage du cancer destinée aux proches de patients atteints de mélanome

Le mélanome est, en partie, un cancer familial

Un de vos proches (frère ou soeur, enfant, père ou mère) a eu un mélanome qui est un cancer de la peau, vous avez un risque plus élevé que la population générale de faire un mélanome à votre tour, **même s'il n'y a qu'un seul cas dans votre famille proche**.

Il vous est recommandé de ce fait
- de vous protéger du soleil
- de réaliser un dépistage annuel du mélanome.

Protection solaire

L'exposition solaire prolongée (plus d'une heure à la plage ou à la piscine, ou en travaillant au soleil ou en faisant du sport), les coups de soleil intenses, favorisent la survenue de mélanome.
S'exposer moins diminue vos risques de mélanome.
Il vous est recommandé:
- de vous exposer le moins possible, en évitant la période de 12h à 16h
- en cas d'exposition, d'utiliser une protection contre le soleil en portant vêtements (T-shirt, casquette ou chapeau) et par crème solaire de très haute protection (indice 50) . L'application de crème solaire est à renouveler toutes les 2 heures.

Dépistage

Vous devez être particulièrement vigilant.
Examinez votre peau tous les 3 mois et consultez un dermatologue une fois par an.
Un mélanome peut ressembler à un grain de beauté.
L'apparition d'un nouveau grain de beauté ou la modification d'un grain de beauté existant doit vous alerter.
L'apparition sur votre peau d'une lésion inhabituelle, et qui persiste plus que 15 jours, doit aussi vous inciter à consulter votre médecin ou votre dermatologue.

Sun Protection and Cancer Screening Tip Sheet for relatives of patients with melanoma

Melanoma is, in part, a family cancer

If one of your relatives (brother or sister, child, father or mother) has had melanoma, which is cancer of the skin, you have a higher risk than the general population of getting melanoma yourself, **even if there is only one case in your immediate family**.

Therefore, it is recommended that you
- protect yourself from the sun
- undergo annual screening for melanoma

Protect yourself from sun exposure

Prolonged sun exposure (more than 1 hour at the beach or pool, working in the sun, or playing sports) and intense sunburn promote the occurrence of melanoma.
Less exposure reduces your risk of melanoma.
Therefore, it is recommended that you
- expose yourself to the sun as little as possible, avoiding the period from 12 noon to 4 p.m.
- in case of exposure, use protection against the sun by wearing clothing (T-shirt, cap or hat) and very-high-protection sunscreen (factor 50)
- renew the application of sunscreen every 2 hours

Screening

You must be particularly vigilant.
Examine your skin every 3 months and see a dermatologist once a year.
A melanoma can look like a mole.
The appearance of a new mole or the modification of an existing mole should be a concern.
The appearance on your skin of an unusual lesion, one that persists for more than 15 days, must also alert you to see your doctor or dermatologist.

Table S1. Socio-demographic characteristics and melanoma risk factors of index cases at inclusion by randomization group

	**Intervention group** Written and oral information n = 60	**Control group** Oral information alone n = 48
Age, n_i=60, n_c=48	63 (15)	61 (15)
Men, n_i=60, n_c=48	31 (51.7)	23 (47.9)
Breslow thickness, n_i=60, n_c= 48	1.3 (0.4 ; 2.7)	2.1(1.1 ; 3.4)
Fitzpatrick skin type		
I	12 (21.8)	6 (12.5)
II	20 (36.4)	25 (52.1)
III	21 (38.2)	17 (35.4)
IV	2 (3.6)	0 (0.0)
Number of nevi > 50, n_i=56, n_c=48	15 (26.8)	13 (27.1)
Histological type, n_i=60, n_c=48		
SSM	45 (75.0)	30 (62.5)
NM	7 (11.7)	7 (14.6)
ALM	4 (6.7)	2 (4.2)
Other	4 (6.7)	9 (18.8)
Ulceration, n_i=60 n_c=48	15 (25.0)	16 (33.3)
History of cutaneous melanoma, n_i=60, n_c=48	3 (5.0)	4 (8.3)
Number of cutaneous melanoma cases, n_i=3, n_c=4	1 (1 ; 1)	1 (1 ; 2)
History of another cancer, n_i=60, n_c=48	6 (10.0)	4 (8.3)
Skin cancer*, n_i=6, n_c=4	3 (50.0)	1 (25.0)
Other type of cancer*, n_i=6, n_c=4	4 (66.7)	4 (100.0)
Family history of melanoma, n_i=60, n_c=47	11 (18.3)	9 (19.1)
Number of people involved, n_i=11, n_c=9	1 (1 ; 1)	1 (1. 1)
Family history of cancer, n_i=60, n_c=46	24 (40.0)	21 (45.7)

Data are n (%), mean (SD) or median (Q1 ; Q3); *a patient could have had several cases of cancer; n_i, intervention group; n_c, control group; SSM, superficial spreading melanoma; NLM, nodular lentiginous melanoma; ALM, acral lentiginous melanoma
